# Three methods for inoculation of viral vectors into plants

**DOI:** 10.3389/fpls.2022.963756

**Published:** 2022-08-30

**Authors:** Andrea G. Monroy-Borrego, Nicole F. Steinmetz

**Affiliations:** ^1^Department of NanoEngineering, University of California, San Diego, San Diego, CA, United States; ^2^Department of Bioengineering, University of California, San Diego, San Diego, CA, United States; ^3^Department of Radiology, University of California, San Diego, San Diego, CA, United States; ^4^Center for Nano-ImmunoEngineering, University of California, San Diego, San Diego, CA, United States; ^5^Moores Cancer Center, University of California, San Diego, San Diego, CA, United States; ^6^Institute for Materials Discovery and Design, University of California, San Diego, San Diego, CA, United States

**Keywords:** plant viral vector, viral nanoparticle, molecular farming, tobacco mosaic virus, transient expression

## Abstract

Agriculture is facing new challenges, with global warming modifying the survival chances for crops, and new pests on the horizon. To keep up with these challenges, gene delivery provides tools to increase crop yields. On the other hand, gene delivery also opens the door for molecular farming of pharmaceuticals in plants. However, towards increased food production and scalable molecular farming, there remain technical difficulties and regulatory hurdles to overcome. The industry-standard is transformation of plants *via Agrobacterium tumefaciens*, but this method is limited to certain plants, requires set up of plant growth facilities and fermentation of bacteria, and introduces lipopolysaccharides contaminants into the system. Therefore, alternate methods are needed. Mechanical inoculation and spray methods have already been discussed in the literature – and here, we compare these methods with a newly introduced petiole injection technique. Because our interest lies in the development of plant viruses as immunotherapies targeting human health as well as gene delivery vectors for agriculture applications, we turned toward tobacco mosaic virus as a model system. We studied the effectiveness of three inoculation techniques: mechanical inoculation, Silwet-77 foliar spray and petiole injections. The foliar spray method was optimized, and we used 0.03% Silwet L-77 to induce infection using either TMV or a lysine-added mutant TMV-Lys. We developed a method using a needle-laden syringe to target and inject the plant virus directly into the vasculature of the plant – we tested injection into the stem and petiole. Stem inoculation resulted in toxicity, but the petiole injection technique was established as a viable strategy. TMV and TMV-Lys were purified from single plants and pooled leaf samples – overall there was little variation between the techniques, as measured by TMV or TMV-Lys yields, highlighting the feasibility of the syringe injection technique to produce virus nanoparticles. There was variation between yields from preparation to preparation with mechanical, spray and syringe inoculation yielding 40–141 mg, 36–56 mg, 18–56 mg TMV per 100 grams of leaves. Similar yields were obtained using TMV-Lys, with 24–38 mg, 17–28, 7–36 mg TMV-Lys per 100 grams of leaves for mechanical, spray and syringe inoculation, respectively. Each method has its advantages: spray inoculation is highly scalable and therefore may find application for farming, the syringe inoculation could provide a clean, aseptic, and controlled approach for molecular farming of pharmaceuticals under good manufacturing protocols (GMP) and would even be applicable for gene delivery to plants in space.

## Introduction

Plants are one of the two main sources of food (animals being the other) and with increasing demand for food, pollution in the environment, chemical threats, as well as natural disasters such as droughts and fires, plant engineering is essential for food production. According to a meta-analysis done in 2021, it is estimated that the demand for food will rise by 35–46 percent between 2010 and 2050 (van Dijk et al., 2021). Plant-based expression systems are also an emerging platform for production of life-saving pharmaceuticals (Chung et al., 2021). This concept of molecular farming was introduced in 1986 for the production of human growth hormone (hGH) in transgenic tobacco and sunflowers (Barta et al., 1986), and achieved years later for an IgG_1_ antibody in transgenic tobacco (Hiatt et al., 1989). More recently, the antibody cocktail ZMapp, which was used to treat Ebola patients during the 2014 epidemic, was produced in *Nicotiana benthamiana* by Kentucky BioProcessing, Inc. (Zhang et al., 2014), while Medicago Inc. uses the same plants to produce seasonal influenza vaccines (Ward et al., 2020). Both companies have also produced COVID-19 vaccine candidates: while Medicago produced a virus-like particle (Ward et al., 2021), Kentucky BioProcessing produced a platform vaccine by conjugating the RBD domain to tobacco mosaic virus (TMV; Royal et al., 2021). Molecular farming has grown out of its infancy and demonstrated its relevance for biopharmaceutical manufacturing.

To increase food production and advance molecular farming, there is a need for improved gene delivery methods for plants. Traditional stable genetic transformation is mostly done using plant callus and *Agrobacterium tumefaciens*-mediated or biolistic particle delivery, with the relatively new short palindromic repeats (CRISPR) associated protein 9 (Cas9) technique being explored (Zhu et al., 2020). However, transgenic plant engineering is costly and it requires long time periods to develop a desirable organism (i.e., the timeframe from callus to a developed plant can take months). Other challenges are that transformation yields may be low or result in undesirable variations; in addition, tight regulations of genetically modified organisms (GMOs) limit their scope for application (Landry and Mitter, 2019; Gad et al., 2020; Zhang et al., 2020a). Transient expression offers plant engineering at a lower cost, faster turnaround time and it can bypass regulatory barriers, due to its time limited effect and transgene-free progeny (Kaur et al., 2021). This could be especially important for agricultural purposes – here plants could transiently express genes as needed in response to current circumstances (e.g., pathogens or environmental threats). For example, if a pest was affecting the area, farmers could protect their crops by transient expression aimed to boost the defensive mechanism of plants. Toward this goal, the CRISPR/Cas9 system has been proposed – CRISPR/Cas9 was used in a fully grown cacao tree to enhance the defense mechanism of the plant by deleting the *TcNPR3* gene, which has been reported to repress the plant’s defense response. Leaf tissue from the engineered cacao tree indeed were protected from pathogen challenge as was shown using a pathogen bioassay (Fister et al., 2018). Similarly, enhancement of the plant’s host defense mechanism was achieved through transient expression of *PnSCR82* in *N. benthamiana* – again effectively protecting plants from pathogen challenge (Wang et al., 2022).

There is still room for improvement and advancement of gene delivery methods to pave the way for broad farming and industrial applications. For transient expression, *A. tumefaciens*-mediated gene transfer or viral vectors are often used, where *A. tumefaciens* can be delivered by vacuum-assisted, spray, and syringe agroinfiltration (Zhang et al., 2020a; Torti et al., 2021). Plant viral vectors are delivered also *via A. tumefaciens* or mechanical inoculation (Marillonnet et al., 2004). Alternative methods include gene transfer *via* nanoparticles such as carbon nanotubes, carbon dots or other nanoparticles based on polymers or metals (Liu et al., 2009; Demirer et al., 2019; Kwak et al., 2019; Wang et al., 2020); however, it is not clear whether these synthetic materials are on par with the biological systems in terms of efficiency and efficacy.

The use of these methods varies by application. For foliar administration: vacuum or syringe infiltration are used for *A. tumefaciens.* While for viral vectors and nanoparticles: mechanical, spray or syringe inoculation can be used. For vacuum-infiltration plants are submerged in a bath containing the *Agrobacterium* and hormones to stimulate gene transfer, a vacuum is then applied in an infiltration chamber, this method requires a vacuum unit and a plant that can be easily handled to be placed in this unit. The agrobacteria will transfer a gene cassette for transient expression of the target gene (Gleba et al., 2013). For syringe-based methods, a needleless syringe is used to infiltrate the leaves directly with a suspension of agrobacteria containing hormones to activate gene transfer (Gleba et al., 2013). For mechanical inoculations, typically viral vectors are gently rubbed onto leaves dusted with carborundum to induce lesions and enable symplastic delivery (Hull, 2009). For foliar spray applications, surfactants such as Silwet-77 are used to reduce tension and allow the active ingredients to transport *via* the waxy cuticle into the leaves *via* pores and stomata followed by symplastic or apoplastic uptake (Hu et al., 2020a). Surfactants have also been used for floral dip (O’Callaghan, 2016).

In this work, we compare mechanical vs. Silwet-77 spray vs. a newly-introduced vascular syringe inoculation method – we used TMV as a model system. TMV was chosen because it is used as a viral vector (Kagale et al., 2012), as well as a nanoparticle platform for vaccine design and drug delivery (Chung et al., 2020). We only considered TMV as a model system in this work, because our primary interest lies in the production and development of viral nanoparticle systems for vaccine and immunotherapy applications (Cai et al., 2019; Shin et al., 2020; Ortega-Rivera et al., 2021). Furthermore, while agrobacterium-based transformation is the gold-standard for transient expression of pharmaceutical proteins, we did not consider it here for the following reasons: the gram-negative bacteria introduce lipopolysaccharides (LPS) into the otherwise LPS-free plant production system. LPS must be removed through additional purification steps to meet safety requirements by the FDA. For plant virus nanoparticles removal of LPS can be challenging; it has been hypothesized that LPS is bound to the interior and exterior surfaces of the virion. In previous work we established LPS removal strategies, however multi-step methods were used, therefore significantly lowering yields (Wang et al., 2019). Another limitation for the agrobacteria-based system is that, both bacteria and plants need to be produced, which makes the system more cumbersome.

**Figure 1:**
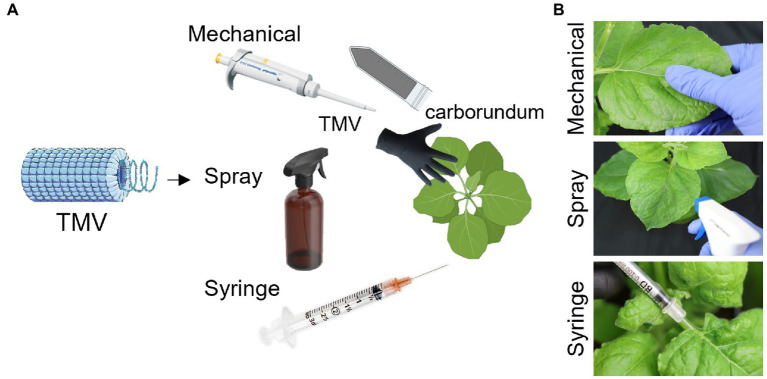
Schematic representation of the experimental design. TMV was used as a model to test mechanical, spray, and syringe inoculation **(A)**. Photographs demonstrating the applications of TMV *via* mechanical, spray, and syringe inoculation **(B)**. The plants from BioRender.com.

Using TMV as a model system, we compared mechanical vs. Silwet-77 foliar, vs. needle-laden injection into the stem or petiole. While spray inoculation is a scalable application and may be useful for in-field applications for farming – the syringe inoculation could provide a clean aseptic controlled approach for molecular farming of pharmaceuticals under good manufacturing protocols (GMP). The method may also find interest if or when molecular farming is introduced into outer space (McNulty et al., 2021). We used *Nicotiana benthamiana* as the model plant, because its widely used as a ‘bioreactor’ in molecular farming. The three methods of inoculation were performed side-by-side and production yields of TMV were determined. The schematics for the experimental design can be seen in Figure 1.

## Materials and methods

### *Nicotiana benthamiana* growth and inoculation with TMV

*N. benthamiana* seeds were planted in Pro Mix BX soil (Greenhouse Megastore) and grown in an A1000 chamber (Conviron) with light at ~100,000 lux, 50–60% humidity, and at 25°C. After 2 weeks the seedlings were transferred to larger pots, and fertilizer was administered once per week (Jack’s Fertilizer #77840, JR Peters Inc.). When the plants were 4–5 weeks old, the inoculations were performed as detailed below. After visual inspection and confirmation of symptoms, leaves were harvested 10–15 days post inoculation and stored at −80°C until further processing. Inoculations were carried out *via* mechanical inoculation of carborundum dusted leaves, Silwet-77 assisted spray (0.02–0.04%), and direct injection into the stem and petiole. 3, 15 and 30 μg of purified TMV in 10 mm sodium phosphate (NaPB) buffer pH 7.4 was used, with 10 plants per treatment. Photographic documentation was carried out on days 0, 8, 10, and 15 post inoculation of TMV. In our studies we used native TMV as well as a Lys-added mutant of TMV, denotated as TMV-Lys described in Geiger et al., 2013.

#### Mechanical inoculation of TMV

Carborundum (C192-500, Thermo Fisher Scientific) was gently dusted on three leaves, and subsequently rubbed by hand with 100 μl of TMV (0.01 mg/ml, 0.5 mg/ml, 0.1 mg/ml) and NaPB buffer pH 7.4 to deliver 3 μg, 15 μg or 30 μg of TMV. Gloves were changed between each plant to avoid carryover of infectious material. Plants were kept in the dark for 1 h post treatment (to avoid burning of carborundum under the grow lights), then were rinsed with tap water and placed into the plant growth chamber.

#### Spray inoculation of TMV

A trigger/spray nozzle (3,345, Control Company) as shown in Figure 1B was used to infect the plants. Three spraying applications were done with each one being ~880 μl of different concentrations of TMV in NaPB buffer pH 7.4 with Silwet L-77 at varying concentrations of 0.04, 0.03, and 0.02% by volume. TMV at 0.0034 mg/ml, 0.017 mg/ml, and 0.0341 mg/ml was used to deliver 3 μg, 15 μg or 30 μg of TMV. After injection plants were immediately placed into the growth chamber – there was no need to rinse.

#### Syringe inoculation of TMV

Insulin syringes with a needle of 8 mm × 31 G (328,438, BD Medical Device Company) were used, and loaded with 2 μl of 1.5, 7.5, or 15 mg/ml TMV in NaPB buffer pH 7.4 to deliver 3 μg, 15 μg or 30 μg of TMV. The loading site for the syringe was tested in the stem, and petiole. After injection plants were immediately placed into the growth chamber – there was no need to rinse.

### Extraction and purification of TMV

The extraction and purification of TMV and TMV-Lys was done as described by Bruckman and Steinmetz (2014). In brief, the leaves were homogenized in NaPB buffer using a commercial blender (6812-001, Oster), this mixture was then filtered through cheesecloth (NC9442780, Fisher Scientific), to then be centrifuged (Beckman Coulter Avanti ® J-E centrifuge, 11,000 *g* for 20 min at 4°C). The supernatant was then filtered through Kimwipes (21905–011, VWR), and mixed with equal parts chloroform/butanol (AC423550040/ A399-4, Fisher Scientific) and mixed for 30 min at 4°C. Then this mixture was centrifuged (Beckman Coulter Avanti ® J-E centrifuge, 4,500 g for 10 min at 4°C), and the top aqueous layer containing the plant virus was taken for the next steps. The viral particles were then precipitated using 8% (w/v) PEG (MW 8000 Da) and 0.2 M NaCl (BP233-1 and BP358-212, Fisher Scientific); this mixture was then placed in a shaker overnight at 4°C. The solution was then centrifuged (Beckman Coulter Avanti ® J-E centrifuge, 22,000 *g* for 20 min at 4°C), and the pellet was then resuspended in 0.1 M NaPB buffer pH 7.4. Followed by another short centrifugation (Beckman Coulter Avanti ® J-E centrifuge, 9,000 *g* for 15 min at 4°C), the supernatant was then ultracentrifuged (Beckman Coulter Optima™ L-90 K centrifuge, 160,000 *g* for 3 h at 4°C) over a 40% sucrose cushion (S0389, Sigma-Aldrich). The pellet was then left on a shaker overnight to resuspend in 10 mm NaPB buffer pH 7.4. For the final step, the solution is passed through a silica column (17-0851-01, Cytiva).

### TMV characterization

To validate the purity and structural integrity of purified TMV, UV-vis spectroscopy, SDS-PAGE, and transmission electron microscopy (TEM) was performed.

#### UV–vis spectroscopy

TMV concentration was determined by UV-vis spectroscopy using a NanoDrop Spectrophotometer (Thermo Fisher Scientific) and Beer-Lambert law with the extinction coefficient of TMV at 260 nm of 3 ml mg^−1^ cm^−1^.

#### Sodium dodecyl sulfate–polyacrylamide gel electrophoresis

For the sample preparation, 2 μg of TMV from the original purified solution were diluted to a final volume of 15 μl with NaPB buffer pH 7.4, to which 4 μl of the 4x lithium dodecyl sulfate (LDS) loading dye (Life Technologies) were added. This solution was then denatured for 5 min at 95°C, and analyzed on NOVEX NuPAGE 4–12% Bis-Tris gels (Invitrogen) in 1x morpholinepropanesulfonic acid (MOPS) buffer (ThermoFisher Scientific). SeeBlue Plus2 was as molecular standard. The gel was ran at 200 V/120 mA for 40 min. Gels were stained with Commassie Brilliant Blue R-250 and imaged using an AlphaImager system (Protein Simple).

#### Transmission electron microscopy

TMV at a concentration of 0.01, 0.05, or 0.1 mg ml^−1^ was placed on Formvar carbon film coated TEM supports (VWR International) and stained with 2% (w/v) uranyl acetate (Agar Scientific). Images were then taken with a FEI TecnaiSpirit G2 BioTWIN TEM at 300 kV.

## Results and discussion

### Mechanical, spray, and syringe inoculation protocols were optimized

For mechanical inoculation established protocols were followed. For foliar spray the Silwet L-77 concentration was optimized. Silwet L-77 has been used for vacuum-assisted agroinfiltration at a concentration of 0.1 and 0.03% (Vojta et al., 2015); for agrospray applications Silwet L-77 was used at 0.1% (Hahn et al., 2015). Data also indicate that 0.1% Silwet L-77 was sufficient for nanoparticle delivery to tomato plants (Zhang et al., 2020b), while 0.2 and 0.3% were required for nanoparticle delivery to cotton and maize (Hu et al., 2020). Based on these data points, 0.02–4% Silwet L-77 spray was applied to 4–5 weeks old and 6–7 weeks old *N. benthamiana* plants. Using 4–5 weeks old plants and sham inoculations, we noted that 24 h post surfactant exposure, concentrations higher than 0.04% indicated tissue damage which was evident by leaf discoloration or darkening of the leaves; higher surfactant concentrations (>0.4%) resulted in necrotic tissue. We noted that plant age plays a role with older plants (6–7 weeks) being more robust and less necrosis observed at higher surfactant concentrations (0.4% Silwet L-77, Supplementary Figure S1). For TMV infection, we tested inoculation using 0.02 and 0.03% Silwet-L77, however only foliar spray inoculation of TMV in presence of 0.03% Silwet-L77 yielded visible symptoms (data not shown), therefore this surfactant concentration was used in all other experiments.

**Figure 2:**
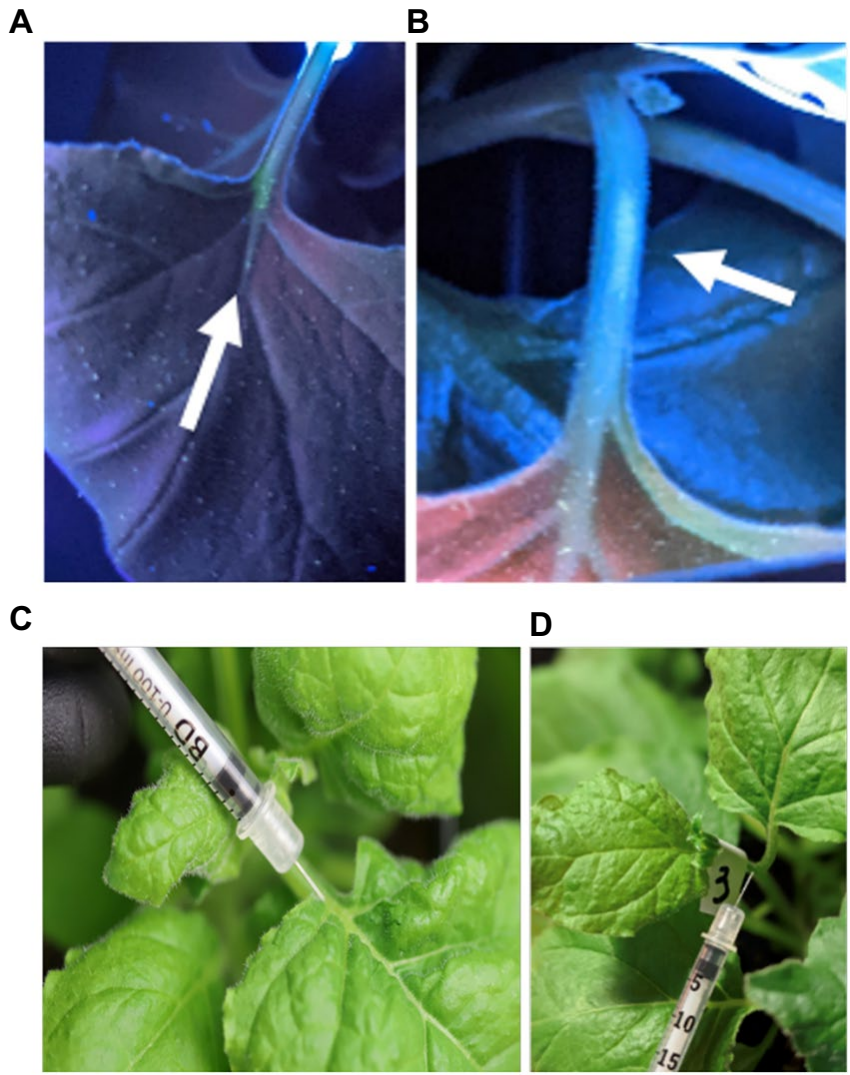
Photographic documentation of the injection of fluorophore Oregon Green 488TM into the petiole; plants were imaged under UV light **(A,B)**. Photograph of TMV injection into the petiole **(C,D)**.

**Figure 3:**
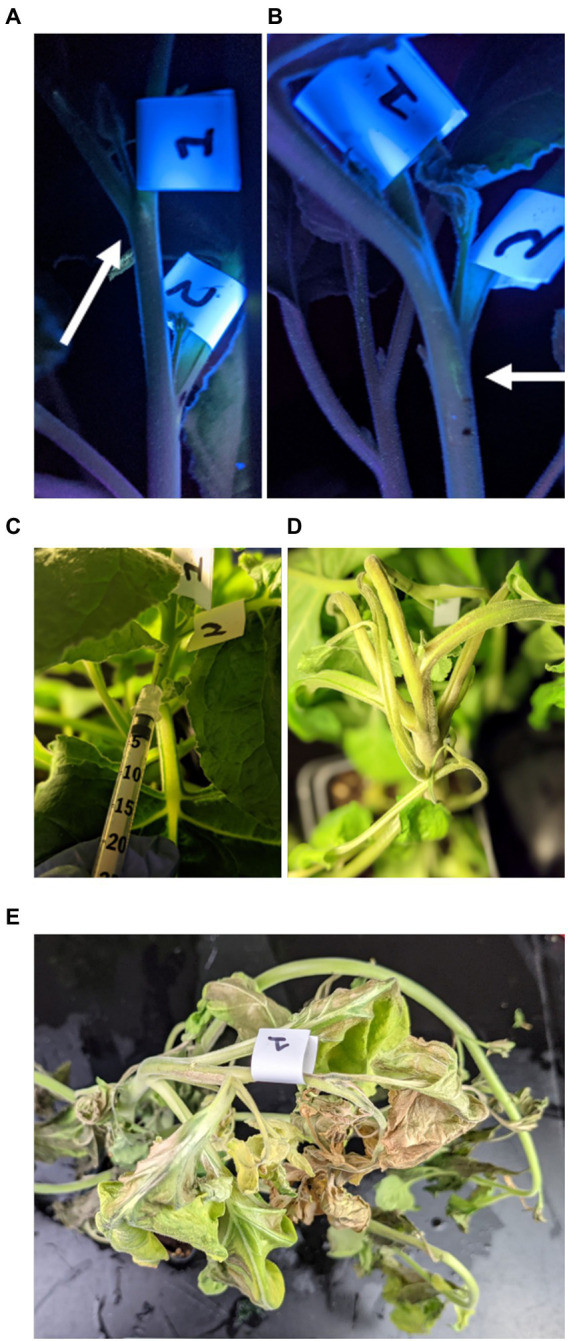
Photographs after injection of the fluorophore Oregon Green 488^™^ in the plant’s stem; the plant was imaged under UV light **(A,B)**. Photographic documentation of TMV injection into the stem **(C)**. After the inoculation, early signs of necrotic tissue were observed in the stem, on average 8 days post infection **(D)**, which would expand causing leaf necrosis and death **(E)**. Therefore, viral syringe inoculation into the stem is not a suitable method for an infectious vector – it may be suitable for non-infectious nanoparticles.

**Figure 4:**
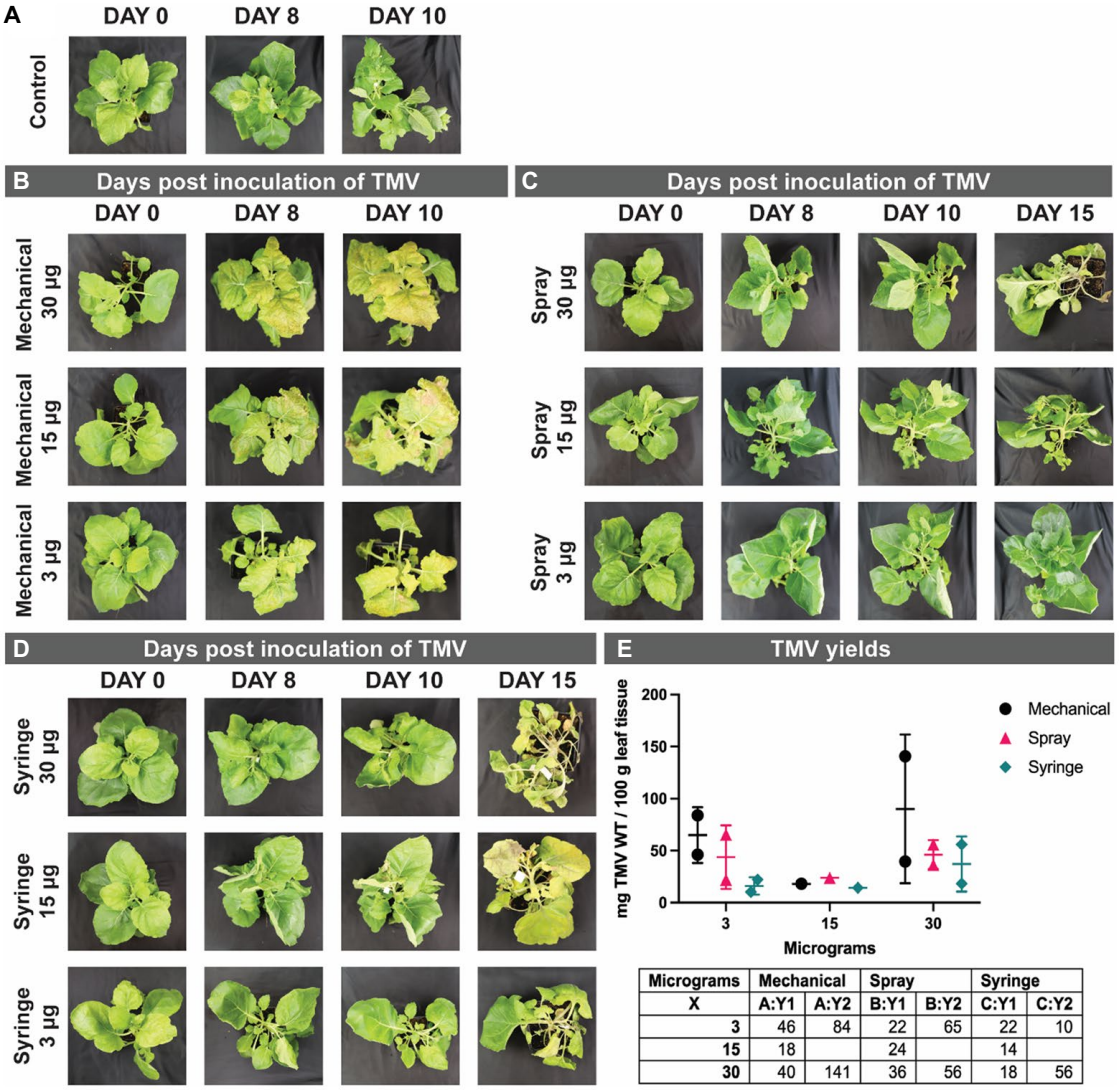
Representative photographs of *N. benthamiana* from day 0 to day 10–15, control non-infected plants **(A)**, and TMV days post inoculation (dpi) by mechanical **(B)**, spray **(C)**, and syringe **(D)** method using 3, 15, and 30 μg of TMV. Graphed yields, and a table of the values for the TMV yields obtained **(E)**. The experiments were done in duplicate using 10 plants per treatment. Additional control plants are shown in the Supplementary Information, Supplementary Figure S3.

**Figure 5:**
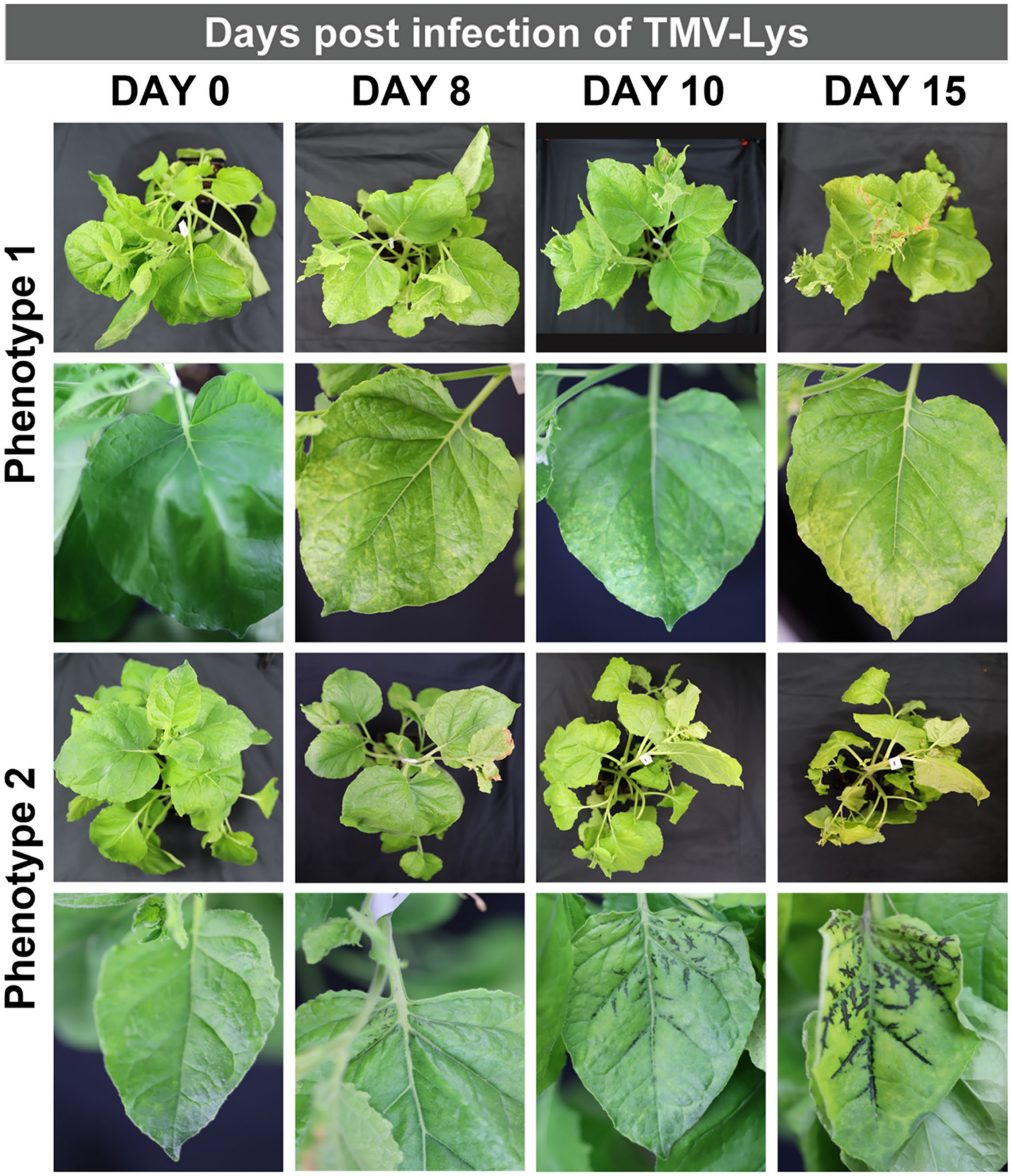
Representative symptoms of *N. benthamiana* infected with TMV-Lys. Phenotype 1, shows generalized yellow mottling of the leaves increasing with time. Phenotype 2, shows the blackening of veins with the simultaneous yellowing of the leaves.

For the vascular syringe method, we assessed feasibility to administer the viral vector into the stem and petiole. As a first testbed, we delivered the fluorophore Oregon Green 488™ to track the injected solution in the plant through imaging under UV light (Figures 2A,B, 3A,B). TMV administration into the petiole resulted in systemic infection (see Figures 4, 5). While, dye or TMV administration into the petiole did not cause adverse effects (Figures 2C,D), injection into the stem resulted in necrotic tissue observed ~8 days post inoculation (dpi) and resulting in systemic necrosis and plant death (Figures C–E). With this knowledge, the petiole was selected as the optimized injection site.

### Distinct phenotypes of TMV infection post mechanical, spray and syringe inoculation method

To demonstrate robustness of the methods, we inoculated plants first with TMV and then repeated the experiments using a lysine-added mutant of TMV, TMV-Lys. Photographic documentation of the TMV-infected plants is shown in Figure 4 and TMV-Lys plants were shown in Supplementary Figure S2 and Figure 5. The main phenotype for TMV infection includes the mosaic/mottling pattern, necrosis, uneven coloring, yellowing, and curling of leaves; however, other symptoms such as blackening of the veins have also been reported (Scholthof, 2004). In our studies, we observed distinct symptoms as a function of the inoculation method.

For mechanical inoculation, local symptoms appeared at 8 days post inoculation (dpi) and systemic infections were established 10+ dpi. Representative photographs are shown in Figure 4B. The most prevalent symptom observed upon mechanical inoculation were the mosaic/mottling patterns, yellowing of leaves, and the presence of necrotic tissue. The severity of the symptoms was higher when plants were inoculated using 30 μg vs. 3 μg of TMV.

In contrast to mechanical inoculation, spray inoculated plants showed symptoms at day 10 (2 days later), and at 15+ dpi systemic infection was reported. Overall, the slightly delayed timeline to establish gene expression and hence infection for the spray vs. mechanical inoculation is in agreement with other reports: while traditional vacuum infiltration takes an average of 4–7 days for gene expression, gene expression is delayed when delivered *via* agrospray, and gene expression was confirmed 10–14 dpi (Hahn et al., 2015). The prevalent symptoms for spray inoculation were yellowing and curling of leaves (Figure 4C). Using higher TMV concentrations (15 and 30 μg), we also noted some plants with blackening of veins and necrotic tissue (not shown).

Syringe inoculation showed TMV symptoms at day 10, and systemic infection at day 15. Diverse TMV symptoms were observed with two main two phenotypes: mosaic/mottling symptoms and blackening of the veins (Figure 4D) – the latter symptoms were also observed for TMV-Lys when plants were inoculated by syringe injection into the stem (Figure 5). For the treatments inoculated with 15 and 30 μg of TMV, the symptoms in the plants were clear, however, for 3 μg of TMV the symptoms passed almost unnoticed. The most significant difference between the various inoculation methods was the degree of variation as to whether or not systemic infection was established. While most plants showed systemic infection by 15 dpi, in some plants TMV infection was not established. Data report that TMV requires phloem loading to establish systemic infection (Cheng et al., 2000) – therefore it is likely that in plants that lacked TMV infection the injection missed the phloem. Therefore, there is room to further innovate this injection method by use of precision needles to target the phloem directly.

Several controls were considered: control plants were cultivated side-by-side in the same growth facility, but in a separate incubator; these plants showed no TMV infection symptoms and are shown in Figure 4A. In addition, for each treatment a control was performed as follows: for mechanical inoculation plants were rubbed with carborundum without TMV, for spray inoculation the 0.03% Silwet L-77 was applied in NaPB buffer pH 7.4, and for the syringe inoculation NaPB buffer pH 7.4 was injected. Infection or symptoms were not apparent, and this data is shown in Supplementary Figure S3.

### The yields for mechanical, spray and syringe inoculation are not statistically different

**Figure 6:**
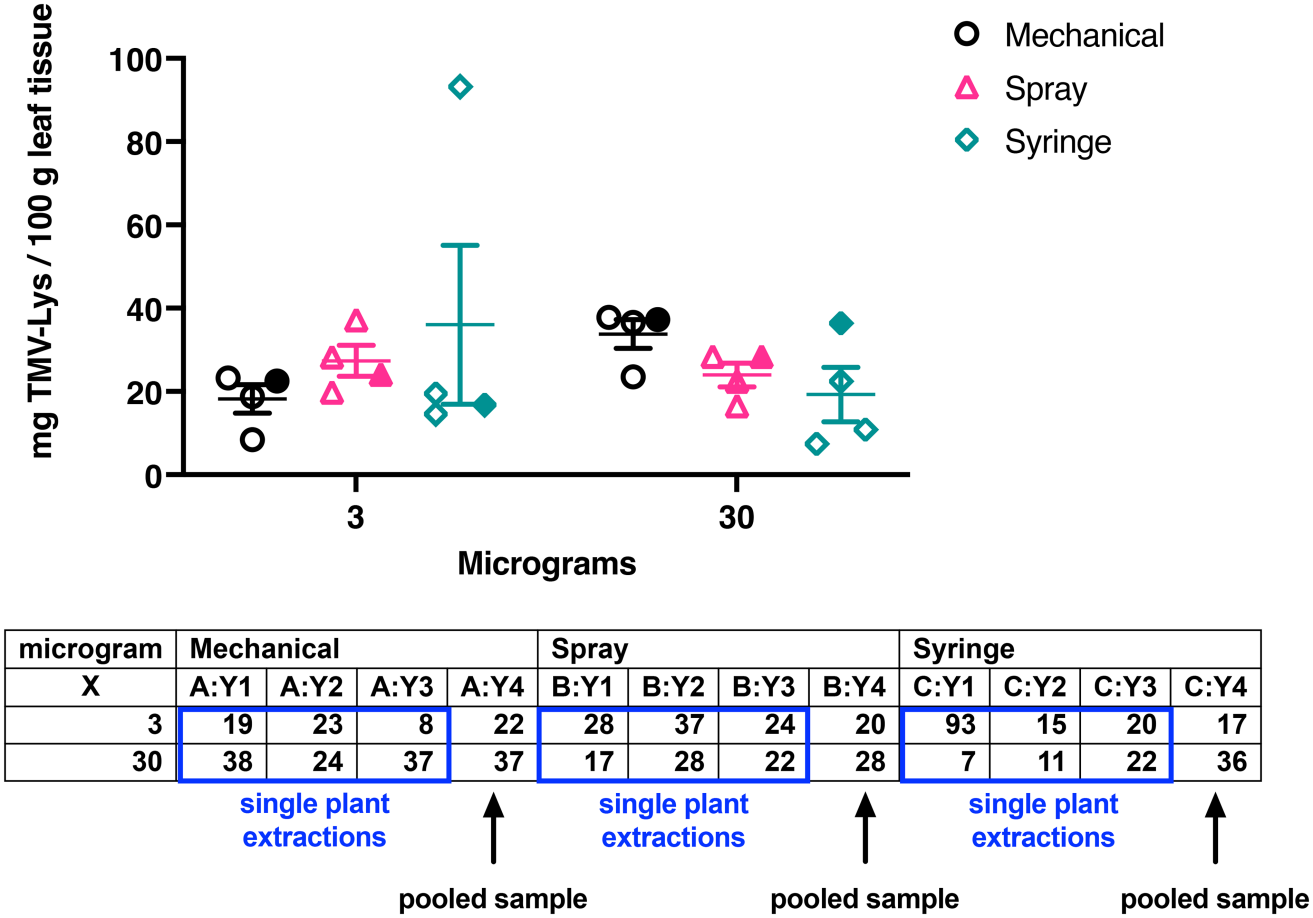
Graph and table showing the yields of TMV-Lys by the different inoculation methods (mechanical, spray, syringe) using 3 or 30 μg of the plant virus. For groups Y1, Y2, and Y3 the data was obtained by extracting TMV-Lys from a single plant (open symbols) – the yields were normalized to 100 grams of leaves for comparison with pooled samples (Y4, filled symbols). Group Y4 are yields from pooled leaves from 10 plants. Data is in good agreement regardless of the sample size.

For TMV harvest, all visibly infected leaves were collected. While there was some variation between the yields comparing the three inoculation methods, there was no statistical differences between the methods (Figure 4E – TMV; Figure 6 – TMV-Lys). We first discuss the TMV data: here leaves were pooled and 100 gram of leaf material was purified: data indicate comparable yields with a trend of increased yield when mechanical inoculation is performed at higher dose: mechanical inoculation using 3 μg vs. 30 μg TMV yielded 46 vs. 90 mg TMV per 100 g of infected leaf tissue – a similar trend was also apparent for the syringe method (16 vs. 37 mg). In contrast there was no dose dependence for spray inoculation using TMV yielding 43–46 mg TMV per 100 g of infected leaf tissue (however these experiments were done using a small sample size). These trends are also in agreement with the symptoms observed: overall, mechanical inoculation resulted in more severe symptoms compared to spray inoculation. Syringe-inoculated plants had a high variation of TMV symptoms, with some plants showing a severe infection while others had only mild symptoms. While we did not set out to study the number of leaves that were infected per plant as a parameter in our studies, no noticeable differences were observed.

A similar trend was observed when TMV-Lys was used: here we compared the yields of TMV-Lys per 100 grams of infected leaves using pooled samples, as was done for TMV, but we also performed single plant extractions and then normalized the yields to 100 g leaf tissue. The data comparing single plant extractions vs. pooled leaves are in good agreement (Figure 6). TMV-Lys yields were comparable at either dose yielding 24–28 mg TMV-Lys per 100 g infected leaf tissue. Also, the syringe inoculation resulted in slightly lower but consistent yields of 17–19 mg TMV-Lys per 100 g infected leaf tissue. Only the mechanical inoculation showed a trend of dose-dependency doubling yields at the higher dose (34 vs. 18 mg TMV-Lys per 100 g infected leaf tissue for mechanical inoculation using 30 μg vs. 3 μg TMV-Lys, Figure 6).

Data suggest that there is no dose-dependence when the viral vectors are inoculated *via* foliar spray. A possible explanation is the mechanism of the surfactant. Silwet L-77 application opens up entry *via* the stomata and the cutical pathway, with the stomata being the main entrance (Hu et al., 2020). Hence, infection of TMV in *N. benthamiana* is dependent on the structure of the leaves, i.e., number of stomata, as well as the capacity of the surfactant to open the entry paths. Therefore, it could be speculated that regardless of the amount of TMV available in the surface of the leaf, only a certain amount would enter the intracellular environment of the plant. This is consistent with the low transfection rates reported for plasmid delivery *via* agrospray resulting in expression rates of 0.9–3.5% - in stark contrast, high expression rates have been reported for viral vectors reaching up to 93% efficiency; the latter can be explained by the cell-to-cell and systemic movement of the viral vector (Hahn et al., 2015). Also, the syringe inoculation yields did not appear to be dose-dependent but success of infection was more variable (see above).

### The identity of TMV is consistent for mechanical, spray and syringe method

**Figure 7:**
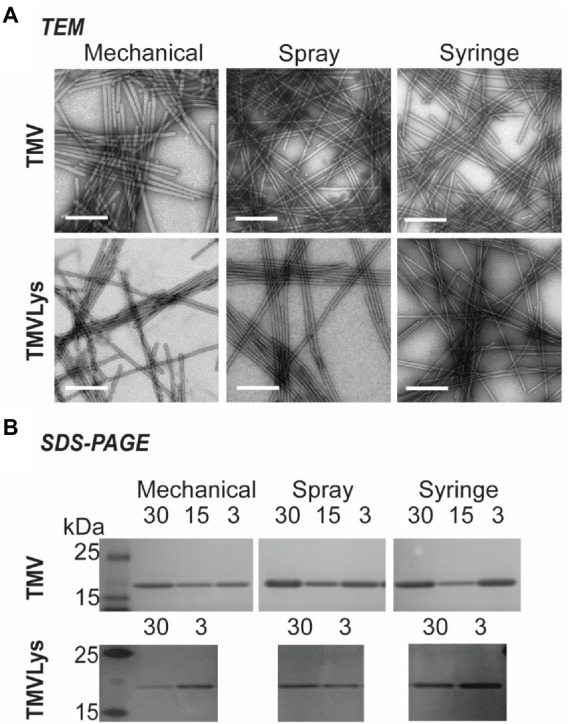
TEM images of TMV and TMV-Lys obtained by different inoculation methods, scale bar represents 200 nm **(A)**. SDS-PAGE of the extracted TMV and TMV-Lys, the presence of its coat protein (17.5 kDa) was consistent in all samples **(B)**.

After TMV was extracted from the plants, UV–vis absorbance, SDS-PAGE and transmission electron microscopy (TEM) imaging was performed to validate the identity of TMV. UV–vis absorbance data indicate an absorbance ratio at 260/280 of 1.2, which is consistent with intact and pure TMV preparations^1^ and values ranged from 1.18 ± 0.03. TEM images show intact TMV with the typical morphology and high aspect ratio (Figure 7A). SDS-PAGE analysis was consistent with the presence of pure TMV preparations showing the 17.5 kDa TMV coat protein; plant contaminants were not apparent; also, contamination of control plants was also not detected (Figure 7B; Supplementary Figure S4).

## Conclusion

In this study, we compared mechanical vs. foliar spray vs. petiole and stem injection of TMV (and TMV-Lys) as a model system for gene delivery. Successful gene delivery was measured by establishment of infection. While injection into the stem of the plant resulted in systemic toxicity and plant loss, targeting the petiole was found productive with good infection rate and yields comparable to any other method. Each of these methods offer advantages – mechanical inoculation shows a high degree of reproducibility given the ease of the method. Foliar spray application is scalable and may offer a broad platform for agricultural engineering and could facilitate transient gene delivery in large extensions of crops. The syringe inoculation provides an aseptic method that may be suitable for the pharmaceutical industry. Compared with the current standard of *Agrobacterium*-based transformation methods, syringe inoculation does not require the culturing of gram-negative bacteria that (i) can be affected by epigenetic variation, (ii) introduce LPS into the product which then requires additional purification steps, and (iii) add complexity to the manufacturing set up, requiring plants and a fermenter. While *Agrobacteria*-based transformation is the effective and currently industry-standard, alternate approaches such as syringe inoculation is an interesting technology to explore. There is room for innovation: infection rate, or gene delivery rate may be improved through use of precision syringes to target the phloem or xylem for a desired application. In fact, the application of microneedles for plant engineering has been suggested (Cao et al., 2020).

## Data availability statement

The original contributions presented in the study are included in the article/Supplementary material, further inquiries can be directed to the corresponding author.

## Author contributions

AM-B and NFS: data analysis, writing and editing, and experimental design. AM-B: experimental work. Both authors contributed to the article and approved the submitted version.

## Funding

This work was supported in part through grants from the NIH (R21-AI161306 and U01 CA218292 to NFS), USDA (NIFA- 2020-67021-31255 to NFS) as well as partially supported by the NSF through the UC San Diego Materials Research Science and Engineering Center (UCSD MRSEC), grant DMR-2011924 (to NFS) and grant CBET-2134535 (to NFS). AM-B is supported by a pre-doctoral fellowship from UC MEXUS CONACYT.

## Conflict of interest

NFS is a co-founder of, has equity in, and has a financial interest with Mosaic ImmunoEngineering Inc., and serves as Director, Board Member, and Acting Chief Scientific Officer, and paid consultant to Mosaic.

The remaining author declares that the research was conducted in the absence of any commercial or financial relationships that could be construed as a potential conflict of interest.

## Publisher’s note

All claims expressed in this article are solely those of the authors and do not necessarily represent those of their affiliated organizations, or those of the publisher, the editors and the reviewers. Any product that may be evaluated in this article, or claim that may be made by its manufacturer, is not guaranteed or endorsed by the publisher.

## Footnotes

^1^ www.dpvweb.net
