# Supplementary material for: Three methods for inoculation of viral vectors into plants
Source: Front Plant Sci. 2022 Aug 30;13:963756. doi: 10.3389/fpls.2022.963756 (PMC9468707; doi:10.3389/fpls.2022.963756)
Supplement: Supplementary file 1 [file Data_Sheet_2.pdf]

Supporting Information for

Three methods for inoculation of viral vectors into plants

Andrea G. Monroy-Borrego[1], Nicole F. Steinmetz[1,2,3,4,5,6*]

[1]Department of NanoEngineering, University of California San Diego, 9500 Gilman Dr., La Jolla, California 92039, United States

[2]Department of Bioengineering, University of California San Diego, 9500 Gilman Dr., La Jolla, California 92039, United States

[3]Department of Radiology, University of California San Diego, 9500 Gilman Dr., La Jolla, California 92039, United States

[4]Center for Nano-ImmunoEngineering, University of California San Diego, 9500 Gilman Dr., La Jolla, California 92039, United States

[5]Moores Cancer Center, University of California San Diego, 9500 Gilman Dr., La Jolla, California 92039, United States

[6]Institute for Materials Discovery and Design, University of California San Diego, 9500 Gilman Dr., La Jolla, California 92039, United States

*Email: nsteinmetz@ucsd.edu

*** Correspondence:**
Nicole F. Steinmetz
nsteinmetz@ucsd.edu

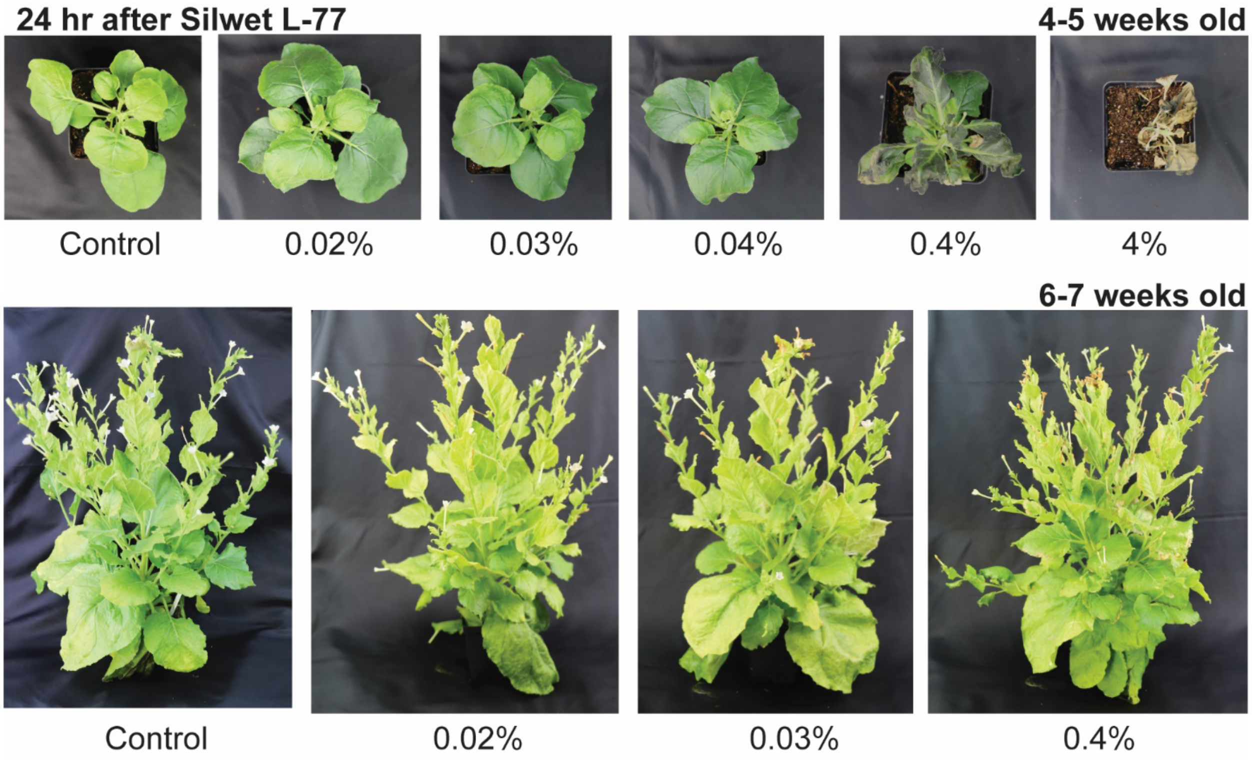

Figure S1. Photographic documentation of the effects of Silwet L-77 spray on *N. benthamiana* 24 hours post treatment. 4-5 weeks old plants tolerated concentrations below 0.04% Silwet L-77. Silwet L-77 treatment caused discoloration and darkening of the leaves which was prevalent at 0.4% Silwet L-77. Using 4% Silwet L-77 caused necrotic tissue. Older plants (6-7 weeks old) were more resistant to the effects of the surfactant, with almost no adverse effects observed for up to a 0.4% of Silwet L-77 (higher concentrations were not tested).

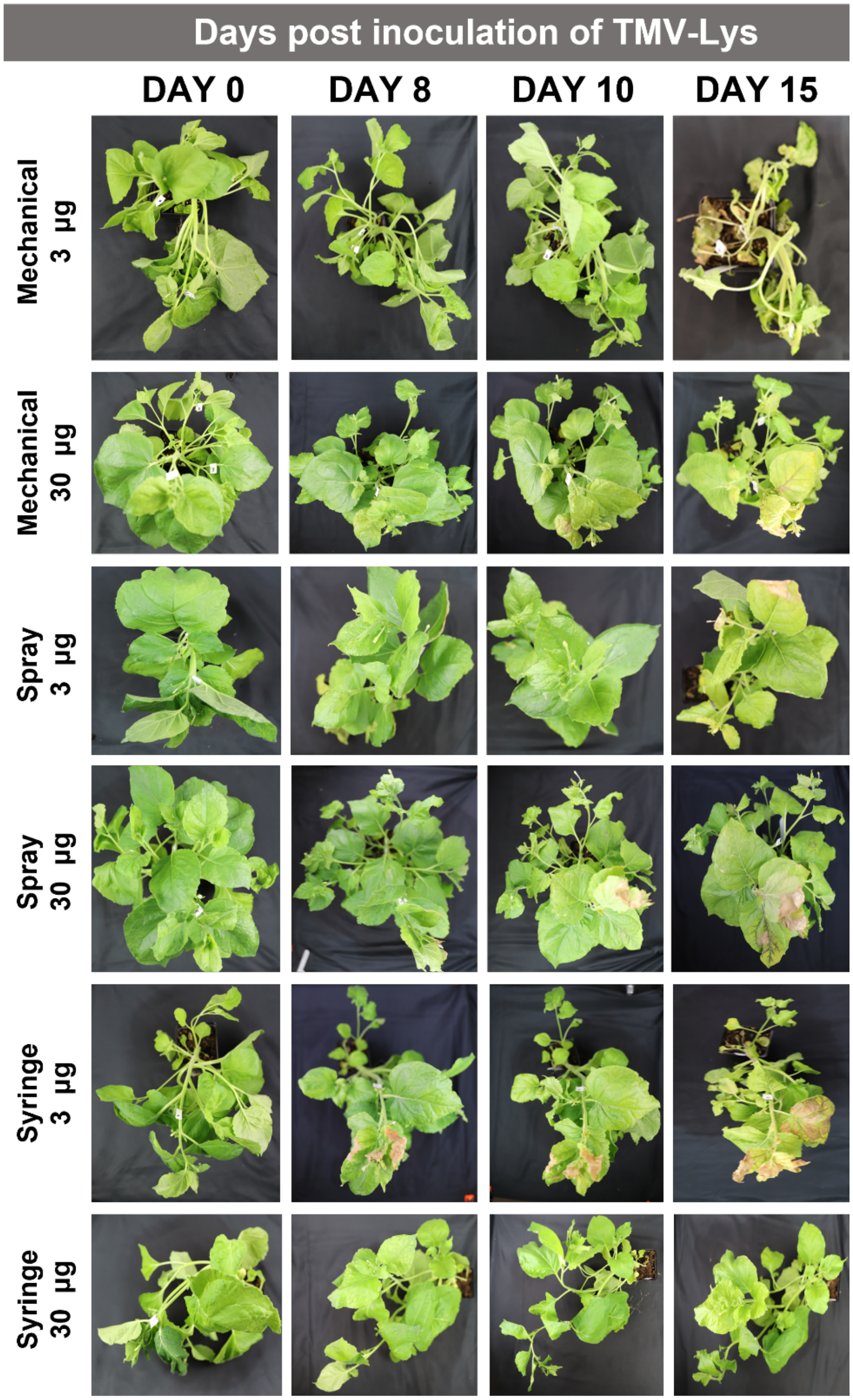

Figure S2. Photographs of *N. benthamiana* from day 0 to day 15 TMV-Lys days post inoculation (dpi) via mechanical [3, 30 µg], spray [3, 30 µg], and syringe [3, 30 µg] inoculation using TMV-Lys. The experiments were done in duplicate and 10 plants were used per treatment.

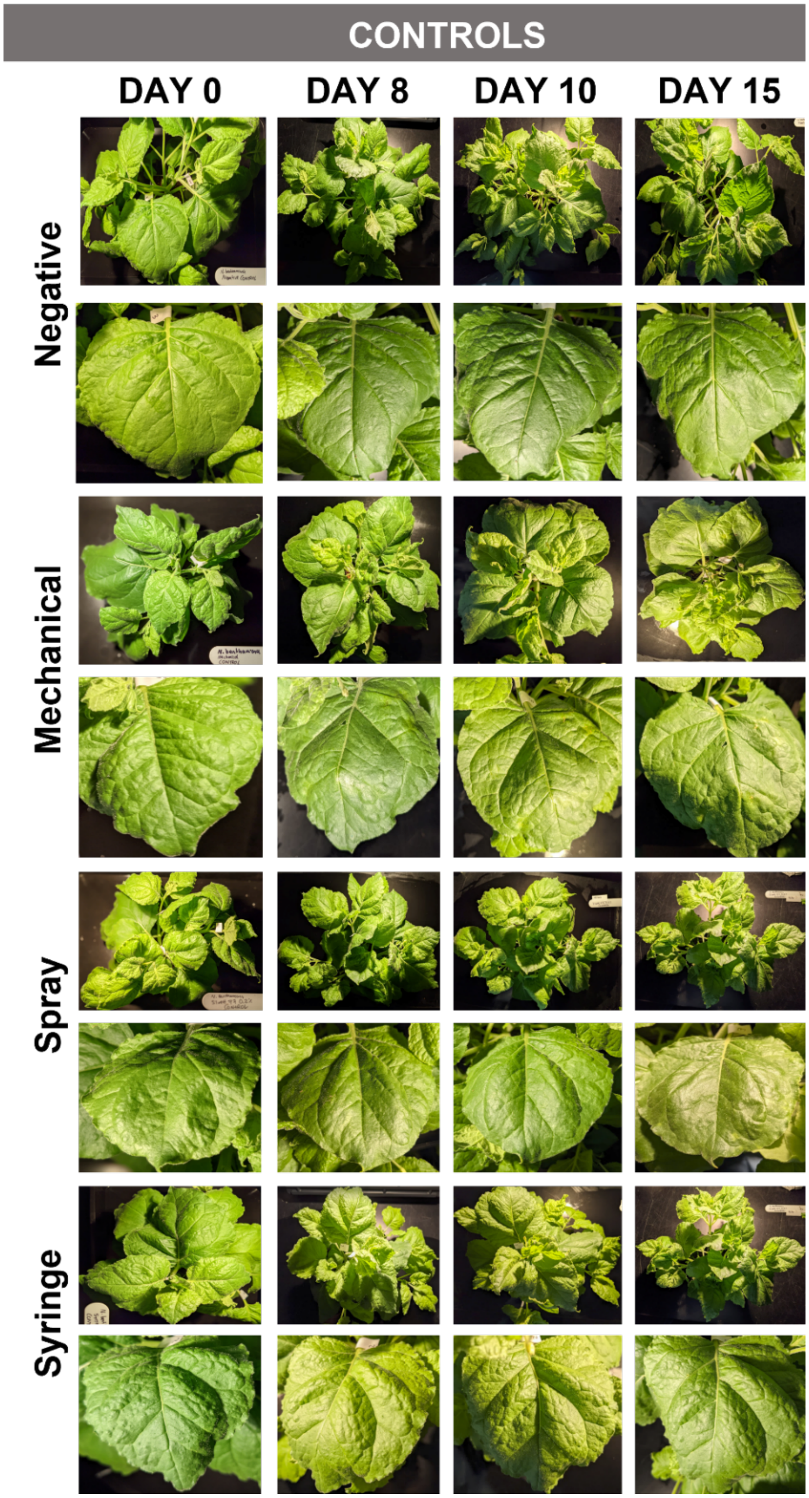

Figure S3. Representative photographs of *N. benthamiana* from day 0 to day 15 post inoculation – these are the negative controls: mechanical control are plants rubbed with carborundum, spray control is treated with 0.03% Silwet L-77, and the syringe control was injected with NaPB at the petiole and stem.

.

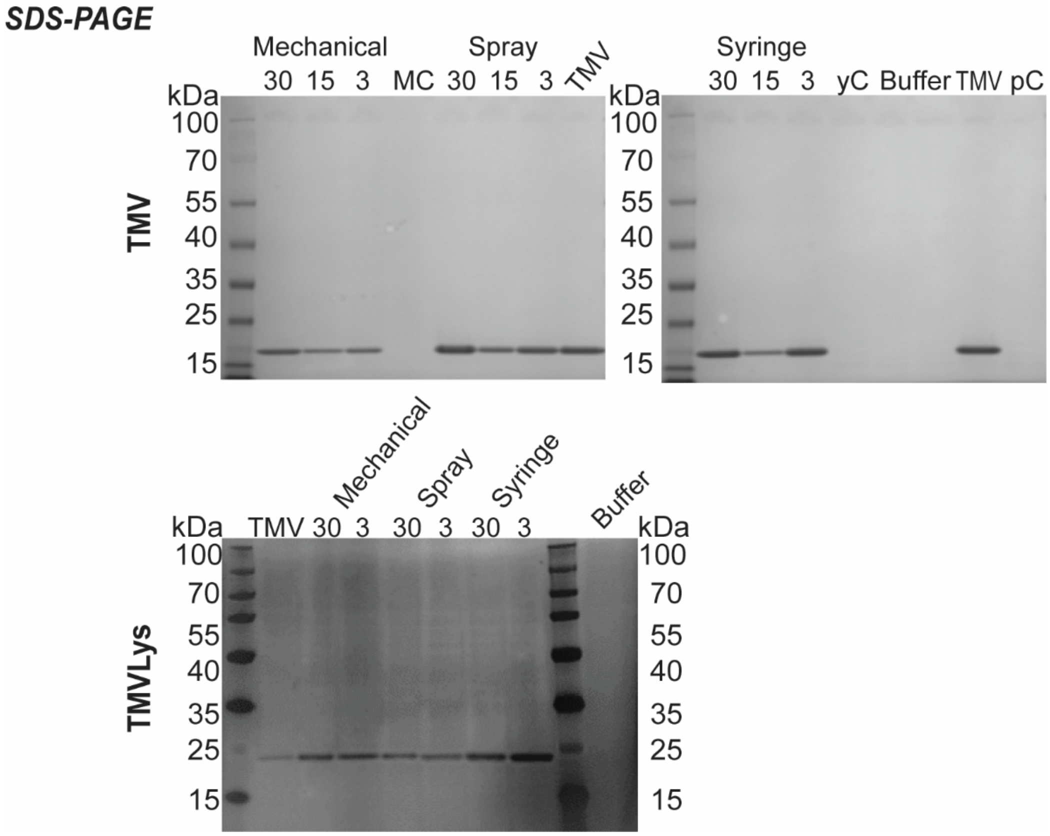

Figure S4. SDS-PAGE of the extracted TMV and TMV-Lys, the presence of its coat protein (17.5 kDa) was consistent in all samples.
